# The potential mediating role of depression on medical mistrust and mental health service use in Black adults

**DOI:** 10.21203/rs.3.rs-6959283/v1

**Published:** 2025-07-15

**Authors:** Aderonke Bamgbose Pederson, Ayla Azman, Jasmin Brooks Stephens, Devan Hawkins

**Affiliations:** Massachusetts General Brigham-Harvard University; University of Washington; University of California, Berkeley; MCPHS University

**Keywords:** medical mistrust, depression, mental health, Black adults, service utilization

## Abstract

**Background:**

Major depressive disorders are ranked third in the global burden of disease by the World Health Organization. The association between mistrust in Black adults and its effect on health service utilization is well established. However, research on mistrust and specific utilization of mental health services is limited. This study examines role of medical mistrust and depressive symptoms on mental health service utilization.

**Methods:**

We conducted an online cross-sectional survey among Black adults (*n* = 1042) using the Group-Based Medical Mistrust Scale, the General Help-Seeking Questionnaire, and the Patient Health Questionaire-9. Gamma regression models were used to assess the relationship between medical mistrust and its association with willingness to use mental health services. We added depressive symptoms to the model to assess whether depressive symptoms may mediate the primary association

**Results:**

Black adults with moderate levels of mistrust reported increasing levels of willingness to seek help (RR = 1.40; 95%CI 1.28, 1.53; p < 0.001 and RR = 1.50; 95%CI 1.38, 1.64; p < 0.001), whereas Black adults at the highest level of mistrust were less likely to seek help from a mental health professional (RR = 1.23; 95%CI = 1.13, 1.34; p < 0.001). The inclusion of depressive symptoms in the model resulted in a 9.5% average decrease in willingness to seek help from a mental health professional.

**Conclusion:**

Depressive symptoms may have a mediating effect on medical mistrust and its association with willingness to seek mental health services for Black adults. Interventions designed to increase service utilization and engagement should consider the role of medical mistrust and the role of depressive symptoms.

## Introduction

Major depressive disorders are among the most common mental illnesses worldwide and ranked third in the global burden of disease by the World Health Organization ^1^. Among Black adults, depression is grossly undertreated due to delayed treatment seeking behavior in this population ^2–4^. Black people are unlikely to receive or seek mental health services compared to their white counterparts ^5^. Racial discrimination in the medical setting is well established and is associated with health care hesitancy (Bazargan et al., 2021; Benkert et al., 2006). Studies show that mitigation of racial discrimination may improve mental health treatment seeking and increase willingness to engage with treatment from mental health professionals ^6^. Mistrust of health systems, in combination with the experience and historical context of racial discrimination, compounds the burden of disease from depressive disorders among racial and ethnic minorities (Cokley et al., 2022; Kim et al., 2017). Understanding the association among medical mistrust, depression, and willingness to engage with mental health services will inform interventions to optimize mental health care engagement for Black adults.

Trust, mistrust, and distrust are distinct concepts in the current literature (Smirnoff et al., 2018). Mistrust does not simply refer to the absence or lack of trust. Rather, medical mistrust is defined as the belief that an individual or system “will not meet an agreed upon expectation or will provide care that does not optimize well-being, or, worse, actively harm a patient,” and trust refers to “Willingness to be vulnerable under conditions of risk and uncertainty,” distrust is closely related to mistrust but is focused on a specific object (e.g., a mental health clinician) ^7^. In this study, we focus on mistrust (i.e., the belief that healthcare workers are actively working against the well-being of the patient) and trust (i.e., the belief that one is treated fairly and equitably within the healthcare system) ^8^. Recent studies show that the burden of mistrust should be placed on the individuals and systems that create inequitable services, rather than on the individual experiencing mistrust ^9,10^. The responsibility to address the root cause of mistrust lies with the institutions and systems that perpetuate conditions that foster mistrust. As such, mistrust may be approached from the perspective of the group experiencing the mistrust or from the group that is responsible for creating the conditions that engender mistrust. Our study focuses on the perspective of one who is experiencing mistrust, and whether mistrust in combination with depression is correlated with willingness to seek mental health services.

There is a known association between mistrust and the use of general health services ^11^; however, there are limited studies on mistrust and specific use of mental health services, and even fewer studies that consider the presence and severity of a disease state like depression. Depression carries a stigmatizing label, and it is well known that stigmatizing illnesses can lead to avoidance of public spaces, including health care institutions ^12–14^. Furthermore, the presence of depression, along with the stigma of depression, and the experience of mistrust can have a compounding effect on the willingness to use mental health services, thereby delaying early engagement in potentially life-saving interventions ^15,16^. In addition, depression can have cognitive impact on executive functioning, slowed processing speed, memory difficulty, and decreased concentration, which can lead to impaired decision-making necessary to organize health care appointments and follow-up visits ^17^.

The correlation between mistrust, depression, and willingness to seek mental health services is important to elucidate. In this study, we aimed to assess the relationship between medical mistrust and trust and their associations with willingness to seek help (for personal and emotional problems and suicidal ideation) from a mental health professional, while considering the role of depression severity. We assessed whether depressive symptoms would moderate the primary association between mistrust/trust and willingness to seek help from a mental health professional. We expected that there would be a negative correlation between mistrust and willingness to seek help from a mental health professional, such that as mistrust increases, we expected willingness to seek help to decrease. We expected the opposite relationship for medical trust. We also hypothesized that higher symptoms of depression would lower help-seeking behavior due to problems of motivation and cognitive slowing. This study contributes to our understanding of how depression symptom severity may impact willingness to engage in service use in the setting of mistrust in a Black adult population.

## Methods

Overview: We conducted an online cross-sectional survey among a cohort of African-Americans, African immigrants, and Afro-Caribbean immigrants (n = 1042) in the United States. We partnered with local community-based organizations (the United African Organization, World Relief Chicago, Coalition for Immigrant Mental Health) to disseminate the survey to their networks. We used convenience sampling to recruit research participants through social media (Twitter and Facebook) on select social media platforms. Eligibility criteria included individuals who: 1) identified as Black, African-American, African or Afro-Caribbean; 2) were 18– 65 years of age; 3) were residing in the United States at the time of the study; and 4) were English-speaking. Written informed consent was obtained from each participant prior to engagement in study activities. Each participant received $25 compensation for their time. BLIND university (IRB ID #: STU00213136) provided IRB approval for study activities.

### Assessments

We report on socio-demographic factors, including age, gender, race, ethnicity, marital status, education, employment status, income, and insurance status.

### The Group-Based Medical Mistrust Scale (GBMMS) ^8^

The GBMMS was designed to assess the tendency to mistrust people or systems outside of one’s racial/ethnic group and the tendency to perceive these systems as fair, equitable, and trustworthy health care entities. Each item is scored on a 5-point Likert-type scale (ranging from “strongly disagree” to “strongly agree”). A principle component analysis of the 12 items revealed two factors that were analyzed: 1) mistrust, 2) trust. Factor 1 (8 items) includes statements about mistrust (negative attitudes towards doctors or health care workers) such as “People of my ethnic group should be suspicious of information from doctors and healthcare workers.” Factor 2 (4 items) includes statements about trust (positive notions), such as “Doctors have the best interests of people of my ethnic group in mind.” Internal consistency for the total GBMMS scale was 0.62. Reliability coefficients for the two factors ranged from 0.83 (Factor 1) and 0.65 (Factor 2).

### General Help-Seeking Questionnaire (GHSQ)

The General Help-Seeking Questionnaire (GHSQ) ^18^: The GHSQ was designed to assess intentions to seek help from 10 different sources (e.g., partner, friend, helpline, or mental health professional) for two different mental health concerns (personal/emotional problems and suicidal ideation). Each item is scored on a 7-point Likert-type scale (ranging from “extremely unlikely” to “extremely likely”). For this study, we used one of the 10 items that assessed intentions to seek help (for personal/emotional problems and suicidal ideation), specifically from mental health professionals such as counselors or psychologists.

### Data Analysis

The frequency and percent of participants according to sociodemographic variables, including age group, gender, marital status, education, annual income, insurance, ethnicity, length of time in the United States, and citizenship status are reported.

We initially examined the relationship between mistrust and willingness to seek help from a mental health professional by calculating mean willingness scores across different values for mistrust. We observed a non-linear relationship with willingness scores increasing as mistrust increased. This relationship was observed both for factors 1 (medical mistrust) and 2 (medical trust). Because of this non-linear relationship, we decided to stratify the sample into four quartiles based on their willingness scores. This stratification was performed for both factors 1 and 2.

We constructed gamma regression models with a log link in SAS Version 9.3 to examine the relationship between mistrust as a predictor of willingness to seek care. For both factors 1 and 2, medical mistrust stratified into four quartiles was treated as a categorical predictor, and willingness to seek care from a mental health professional for (1) a personal or emotional problem and (2) suicidal ideation was treated as a continuous outcome. The rate ratios obtained from these models represented how many times greater or lower willingness to seek care was for quartiles 2, 3, and 4 compared to quartile 1 (the reference group). We constructed univariable models controlling for only mistrust or trust as a predictor and then a multivariable model that controlled for age group, ethnicity, and education. Finally, because we were interested in whether this relationship may be mediated by depression, we also added depressive symptoms to the model (based on the Patient Health Questionaire-9 or PHQ-9 score).

## Results

### Primary Analysis

The association between general medical mistrust and willingness to seek help from a mental health professional (adjusted analysis)

1. After adjusting for age, education, and ethnicity, we examined the association between medical mistrust and willingness to seek help from a mental health professional. Table 2 shows the unadjusted and adjusted analyses. We did not see significant changes in rate ratio between the adjusted and unadjusted models.
  1. Mistrust was associated with willingness to seek help from a mental health professional for personal and emotional problems. Based on the adjusted analysis, respondents with mistrust levels at the 2nd and 3rd quartiles (higher quartiles represent higher levels of mistrust) were 40% and 50% more likely to seek help from a mental health professional (RR = 1.40, p = 0.0001; CI 1.28, 1.53 and RR = 1.50, p = 0.0001; CI 1.38, 1.64). Respondents in the fourth quartile of mistrust (highest level of mistrust) were 23% more likely to seek help from a mental health professional for personal and emotional problems (RR = 1.23, p = 0.0001; CI 1.13, 1.34). Compared to those at the mid-levels of mistrust, those at the highest level of mistrust were less likely to seek help for personal and emotional problems.
  2. Mistrust was also associated with willingness to seek help from a mental health professional for suicidal ideation. Based on the adjusted analysis, respondents with mistrust levels at the 2nd and 3rd quartiles were 36% and 44% more likely to seek help from a mental health professional (RR = 1.36, p = 0.0001; CI 1.24, 1.49 and RR = 1.44, p = 0.0001; CI 1.31, 1.57). Respondents at the fourth quartile of mistrust (highest level of mistrust) were 23% more likely to seek help from a mental health professional for suicidal ideation (RR = 1.23, p = 0.0001; CI 1.13, 1.34). Compared to those at the mid-levels of mistrust, those at the highest level of mistrust were less likely to seek help for suicidal ideation.
  3. Trust was associated with willingness to seek help from a mental health professional for personal and emotional problems. Based on the adjusted analysis, respondents with trust levels at the 2nd and 3rd quartiles were 27% and 25% more likely to seek help from a mental health professional (RR = 1.27, p = 0.0001; CI 1.16, 1.40 and RR = 1.25, p = 0.0001; CI 1.14, 1.37).
  4. Trust was also associated with willingness to seek help from a mental health professional for suicidal ideation. Based on the adjusted analysis, respondents with trust levels at the 2nd and 3rd quartiles were 16% and 13% more likely to seek help from a mental health professional (RR = 1.16, p = 0.002; CI 1.06, 1.27 and RR = 1.13, p = 0.011; CI 1.03, 1.23). Respondents in the fourth quartile of trust (highest level of trust) were 10% less likely to seek help from a mental health professional for suicidal ideation (RR = 0.90, p = 0.024; CI 0.82, 0.99).

### Depressive Symptoms as a Potential Mediator of the primary association

After controlling for ethnicity, education, and age in the association between general medical mistrust/trust and willingness to seek help from a mental health professional, we examined the degree to which depressive symptoms may mediate the primary association by adding depressive symptoms to the model. In the association between medical mistrust and willingness to seek help from a mental health professional, after adding depressive symptoms to the model, there was a decrease by an average of 9.5% in willingness to seek help from a mental health professional. This indicates that 9.5% of the relationship between mistrust and willingness to seek help from a mental health professional may be mediated by depression. For suicidal ideation, after controlling for depressive symptoms, there was a decrease by an average of 5.8% in willingness to seek help from a mental health professional. This indicates that 5.8% of the relationship between mistrust and willingness to seek help from a mental health professional may be mediated by depression.

1.1 In the association between medical trust and willingness to seek help from a mental health professional for personal and emotional problems, after controlling for depressive symptoms, there was a decrease by an average of 4.2% in willingness to seek help from a mental health professional. This indicates that 4.2% of the relationship between trust and willingness to seek help from a mental health professional may be mediated by depression.

For suicidal ideation, after controlling for depressive symptoms, there was a decrease by an average of 3.6% in willingness to seek help from a mental health professional. This indicates that 3.6% of the relationship between trust and willingness to seek help from a mental health professional may be mediated by depression.

### 2. Mean depressive symptom scores based on level of mistrust/trust

As shown in Table 4, respondents who reported the lowest level of mistrust (quartile 1) had a mean depressive symptoms severity of 8.03 (mild depression). Those who reported mid-levels of mistrust (quartiles 2 and 3) had a mean depressive symptom severity of 12.20–13.03 (moderate depression), while those with the highest level of mistrust (quartile 4) had a mean depressive symptom severity of 11.32 (moderate depression). The highest mean score for depressive symptoms was among those in the third quartile.

Respondents who reported the lowest level of trust (quartile 1) had a mean depressive symptoms severity of 9.68 (mild depression), those who reported mid-levels of trust (quartiles 2 and 3) had a mean depressive symptom severity of 12.45–12.85 (moderate depression), while those with the highest level of mistrust (quartile 4) had the same mean depressive symptom severity of those with the lowest level of trust 9.68 (mild depression). The highest mean score for depressive symptoms was among those in the second quartile.

### 3. Depressive symptoms and probability of willingness to seek help from a mental health professional

As shown in Table 5, for every one-unit increase in depressive symptoms, there was a 3.3% increase in the probability of willingness to seek help from a mental health professional for personal or emotional problems (RR = 1.033, p = 0.0001; CI 1.027, 1.039).

For every one-unit increase in depressive symptoms, there was a 2.4% increase in the probability of willingness to seek help from a mental health professional for suicidal ideation (RR = 1.024, p = 0.0001; CI 1.018, 1.030).

## Discussion

The overall aim of the current study was to examine the association between medical mistrust, trust, and willingness to seek help from a mental health professional for personal and emotional problems and suicidal ideation. Furthermore, we aimed to elucidate the role of depressive symptoms on the association between medical mistrust, trust, and willingness to seek help from a mental health professional. This is an understudied area in mental health research literature among an ethnically diverse Black adult population.

In this cross-sectional analysis, we found a nonlinear association between medical mistrust and willingness to seek help from a mental health professional. Specifically, individuals reporting mid-levels of mistrust (i.e., second and third quartiles) reported an increased willingness to use mental health services as compared to those at the lowest levels of medical mistrust. However, individuals at the highest level of medical mistrust (i.e., fourth quartile) were less likely than individuals at mid-levels of mistrust to seek help from a mental health professional. This finding is consistent with results from a previous study, which used this sample pool and found mistrust and trust functioned on a U-shaped gradient, where average levels of mistrust functioned in a mitigating manner and did not prohibit willingness to use mental health services; however, high mistrust was associated with lower use of mental health services ^19^. In this study, we further explore the potential mediating role of depression. High mistrust (fourth quartile) may be associated with under-reporting of mental health concerns ^9,20–22^, which may explain the lower levels of depressive symptoms reported among individuals with high mistrust in our study.

We found a similar nonlinear association between medical trust and willingness to seek help from a mental health professional. Individuals reporting mid-levels of trust (i.e., second and third quartiles) demonstrated a stepwise increasing willingness to seek mental health services for emotional and personal problems and suicidal ideation, as compared to those at the lowest levels of trust. However, respondents at the highest level of trust (i.e., fourth quartile) were less likely than individuals at mid-levels of medical trust to seek help. A potential explanation for this finding may be that individuals with high levels of medical trust may not appraise their level of depressive symptomatology as something for which to seek mental health services (Rickwood et al., 2007).

In this study, we also examined the role of depressive symptoms in the association between medical mistrust, trust, and willingness to use mental health services. Consistent with our hypothesis, depression may have a mediating role in the association between medical mistrust and willingness to use mental health services. To better characterize this potential mediating role, we found that as medical mistrust increased, depressive symptoms increased at the second and third quartiles. The correlation between mistrust and depressive symptoms may be related to the notion that mistrust itself can cause psychological distress, compounding the burden of depression and worsening depressive symptoms (Whaley, 2001). Depressive symptoms can also include hypersensitivity to rejection and increased feelings of mistrust of others, including doctors (Hudson et al., 2018; Pederson, 2023). The highest level of mistrust (fourth quartile) may be associated with under-reporting of depressive symptoms or guarded behavior in help-seeking ^9,20–22^, which may explain the lower levels of depressive symptoms in our study.

We found that the presence of depressive symptoms was correlated with a small increase in help-seeking behavior, and that depressive symptoms explained a portion of the correlation between mistrust and willingness to seek help. This finding is consistent with a past study among Black men, which documented that the majority of participants acknowledged depression as an illness that needed treatment; however, the majority had never sought treatment for depression ^23^. Importantly, our focus on one’s willingness to seek help differs from the actual behavior one might choose to act on (e.g., visiting a counselor). Evidence of hesitancy and mistrust of health care systems has been shown for preventative care among Black men across several studies ^20,24^. In reference to mental health services, while mistrust acts as a barrier to seeking care (Hudson et al., 2018), our study findings expound upon previous research by indicating that the illness itself (depressive symptoms) also has a role in willingness to seek care. Therefore, rather than disaggregating the role of mistrust from the illness itself, we highlight the intersecting role that these two factors (mistrust and depression) may have on mental health service utilization. Our findings support that mistrust, trust, and depressive symptoms serve as a more precise target when designing interventions to increase willingness and change behavior in seeking mental health care for those who need it with active depressive symptoms.

### Study Limitations

Cross-sectional data limits our ability to comment on causal associations, but we were able to identify correlation among variables. We collected data on a convenience sample of 1042 Black adults, which enhances the power of our study. The convenience sample is not representative of the Black adult population in the United States; we had an overrepresentation of male respondents compared to the general population. Despite this limitation, the mental health needs of Black male adults is a high public health priority; Black males have low utilization of mental health services and experience high levels of medical mistrust. Data was collected during the COVID-19 pandemic that may influence how respondents answered questions on their mental health experiences. There are limitations in the generalizability of study findings. Future research is needed to extend our study by using a representative Black adult population, considering experiences across age groups, and applying a longitudinal analysis to engage in causal analysis. Additionally, the current study did not account for various forms of mental health professionals, which may influence help-seeking behavior. For example, religious leaders can serve as the first line of help-seeking when depressive symptoms develop and a referral pathway to mainstream mental health services ^21^.

## Conclusion

Overall, study findings suggest that depressive symptoms may have a mediating effect on medical mistrust and its association with willingness to seek mental health services for Black adults. This study underscores the complex dynamics that affect the pathways towards mental health service utilization among Black adults. The study highlights the importance of addressing medical mistrust and depressive symptoms as key potential mechanistic targets in reducing mental health disparities. It is possible to improve access to and utilization of mental health services within the Black community, ultimately promoting better mental health outcomes by targeting precise factors through appropriate interventions. Willingness to report depression severity may be a factor in those with the highest level of mistrust reporting lower depression symptoms compared to people with moderate levels of mistrust. Whereas for those with trust, there may be high levels of optimism at very high levels of trust that correlate with lower depressive symptoms at the highest levels of trust.

## Figures and Tables

**Table 1:** Descriptive demographics and sample characteristics (N = 1042)

	n	%
Age		
18–44	749	71.9
45–65	293	28.1
Gender		
Male	677	65.0
Female	364	34.9
Other	1	0.1
Marital status		
Married	665	63.8
Unmarried, living with a romantic partner	162	15.6
Never married	139	13.3
Separated	23	2.2
Divorced	37	3.6
Widowed	16	1.5
Education		
Less than high school	29	2.8
High school diploma / GED	147	14.1
Trade school/vocational school	167	16.0
Some college, no degree	280	27.0
2-year college	143	13.7
4-year college degree	217	20.8
Master’s degree	42	4.0
Doctoral degree	17	1.5
Annual income		
$9,999 or less	86	8.3
$10,000 to $29,999	249	23.9
$30,000 to $49,999	349	33.5
$50,000 or more	358	34.4
Insurance		
None	209	20.1
Public	620	59.5
Private	208	20.0
Military healthcare	5	0.5
Ethnic identity/origin		
African	187	18.0
African-American	804	77.2
Afro-Caribbean	44	4.2
Other*	7	0.7
How long in U.S.?		
Less than 2 years	17	1.6
2 to 5 years	142	13.6
6 to 10 years	222	21.3
More than 10 years	661	63.4
Citizenship status		
Citizen	863	82.8
Non-citizen	179	17.2

* Person identified as Black but did not select any of the three ethnicities and was not included in the stratified analysis that measured ethnicity

**Table 2:** Mistrust and willingness to seek help from a mental health professional; Trust and willingness to seek help from a mental health professional

	If you were having a personal or emotional problem, how likely is it that you would seek help from a mental health professional?									
			Univariable				Controlling for age, race/ethnicity, and education			
Factor 1 (mistrust)	Mean	SD	RR	Lower 95% CI	Upper 95% CI	P-value	RR	Lower 95% CI	Upper 95% CI	P-value
Quartile 1	2.39	1.41	1.00	1.00			1.00			
Quartile 2	3.35	1.48	1.40	1.28	1.53	< .0001	1.40	1.28	1.53	< .0001
Quartile 3	3.62	1.59	1.51	1.38	1.65	< .0001	1.50	1.38	1.64	< .0001
Quartile 4	2.93	1.44	1.22	1.13	1.33	< .0001	1.23	1.13	1.34	< .0001
**Factor 2 (trust)**										
Quartile 1	2.95	1.60	1.00				1.00			
Quartile 2	3.49	1.58	1.18	1.08	1.30	0.0004	1.27	1.16	1.40	< .0001
Quartile 3	3.43	1.56	1.16	1.06	1.27	0.0012	1.25	1.14	1.37	< .0001
Quartile 4	2.71	1.54	0.92	0.84	1.01	0.0675	1.09	1.00	1.20	0.054
	If you were having a suicidal ideation, how likely is it that you would seek help from a mental health professional?									
Factor 1 (mistrust)	Mean	SD	RR	Lower 95% CI	Upper 95% CI	P-value	RR	Lower 95% CI	Upper 95% CI	P-value
Quartile 1	2.47	1.46	1.00	1.00						
Quartile 2	3.40	1.59	1.38	1.26	1.51	< .0001	1.36	1.24	1.49	< .0001
Quartile 3	3.59	1.56	1.45	1.33	1.59	< .0001	1.44	1.31	1.57	< .0001
Quartile 4	3.08	1.58	1.25	1.14	1.36	< .0001	1.23	1.13	1.34	< .0001
**Factor 2 (trust)**										
Quartile 1	2.95	1.60	1.00				1.00			
Quartile 2	3.49	1.58	1.18	1.08	1.30	0.0004	1.16	1.06	1.27	0.002
Quartile 3	3.43	1.56	1.16	1.06	1.27	0.0012	1.13	1.03	1.23	0.011
Quartile 4	2.71	1.54	0.92	0.84	1.01	0.0675	0.90	0.82	0.99	0.025

RR = Rate ratio

CI = Confidence Interval

SD = Standard deviation

**Table 3:** Depressive symptoms as a potential mediator of the relationship between mistrust and trust and the willingness to seek help from a mental health professional

			If you were having a personal or emotional problem, how likely is it that you would seek help from a mental health professional?									
			Controlling for PHQ9					Controlling for age, race/ethnicity, education and PHQ9				
Factor 1 (mistrust)	Mean	SD	RR	Lower 95% CI	Upper 95% CI	P-value	Percent change in RR when controlling for PHQ9	RR	Lower 95% CI	Upper 95% CI	P-value	Percent change in RR when controlling for PHQ9
Quartile 1	2.39	1.41										
Quartile 2	3.35	1.48	1.27	1.16	1.39	< .0001	−9.6	1.27	1.16	1.39	<.0001	−9.1
Quartile 3	3.62	1.59	1.34	1.23	1.47	< .0001	−11.3	1.34	1.22	1.47	<.0001	−10.9
Quartile 4	2.93	1.44	1.12	1.03	1.22	0.0082	−8.5	1.13	1.04	1.23	0.0046	−8.4
Average percent change for all factors							−9.8					−9.5
**Factor 2 (trust)**												
Quartile 1	2.59	1.46										
Quartile 2	3.36	1.47	1.2	1.1	1.31	< .0001	−7.4	1.19	1.09	1.3	0.0002	−6.8
Quartile 3	3.35	1.49	1.21	1.11	1.32	< .0001	−6.4	1.18	1.08	1.29	0.0003	−5.5
Quartile 4	2.91	1.63	1.11	1.02	1.21	0.014	−0.9	1.09	1	1.19	0.054	−0.3
Average percent change for all factors							−4.9					−4.2
			If you were having a suicidal ideation, how likely is it that you would seek help from a mental health professional?									
			Controlling for PHQ9					Controlling for age, race/ethnicity, education and PHQ9				
Factor 1 (mistrust)	Mean	SD	RR	Lower 95% CI	Upper 95% CI	P-value		RR	Lower 95% CI	Upper 95% CI	P-value	
Quartile 1	2.47	1.46										
Quartile 2	3.4	1.59	1.29	1.17	1.41	< .0001	−6.7	1.28	1.17	1.4	< .0001	−6.0
Quartile 3	3.59	1.56	1.35	1.23	1.48	< .0001	−7.5	1.34	1.22	1.47	< .0001	−6.8
Quartile 4	3.08	1.58	1.19	1.09	1.29	0.0001	−4.8	1.17	1.07	1.28	0.0004	−4.8
Average percent change for all factors							−6.3					−5.8
**Factor 2 (trust)**												
			If you were having a personal or emotional problem, how likely is it that you would seek help from a mental health professional?									
			Controlling for PHQ9					Controlling for age, race/ethnicity, education and PHQ9				
Factor 1 (mistrust)	Mean	SD	RR	Lower 95% CI	Upper 95% CI	P-value	Percent change in RR when controlling for PHQ9	RR	Lower 95% CI	Upper 95% CI	P-value	Percent change in RR when controlling for PHQ9
Quartile 1	2.95	1.6										
Quartile 2	3.49	1.58	1.11	1.01	1.22	0.0349	−6.5	1.10	1	1.2	0.0539	−5.4
Quartile 3	3.43	1.56	1.09	1	1.2	0.0533	−5.9	1.07	0.98	1.18	0.132	−4.7
Quartile 4	2.71	1.54	0.90	0.83	0.99	0.0245	−1.7	0.89	0.82	0.98	0.0147	−0.7
Average percent change for all factors							−4.7					−3.6

RR = Rate ratio

CI = Confidence Interval

SD = Standard deviation

**Table 4:** Average Depressive symptoms scores by medical mistrust and trust

Factor 1 (mistrust)	Mean	SD	P-value
Quartile 1	8.03	5.21	< 0.0001
Quartile 2	12.20	3.98	
Quartile 3	13.03	4.19	
Quartile 4	11.32	6.43	
**Factor 2 (trust)**			
Quartile 1	9.68	7.28	< 0.0001
Quartile 2	12.85	4.31	
Quartile 3	12.45	4.26	
Quartile 4	9.68	4.99	

SD = Standard deviation

**Table 5:** Relationship between depression and willingness to seek care

	If you were having a personal or emotional problem, how likely is it that you would seek help from a mental health professional?			
	RR	Lower	Upper	P-value
**PHQ-9**	1.033	1.027	1.039	< .0001
	If you were having a suicidal ideation, how likely is it that you would seek help from a mental health professional?			
	RR	Lower	Upper	P-value
**PHQ-9**	1.024	1.018	1.030	< .0001

RR = Rate ratio

CI = Confidence Interval

## Data Availability

Research data is available upon request.
